# Mental imagery in bipolar affective disorder versus unipolar depression: Investigating cognitions at times of ‘positive’ mood

**DOI:** 10.1016/j.jad.2014.05.007

**Published:** 2014-09

**Authors:** Annabel Ivins, Martina Di Simplicio, Helen Close, Guy M. Goodwin, Emily Holmes

**Affiliations:** a Northamptonshire Healthcare NHS Foundation Trust, Northampton, UK; b MRC Cognition and Brain Sciences Unit, Cambridge CB2 7EF, UK; c Oxford Health NHS Foundation Trust, Oxford, UK; d Department of Psychiatry, University of Oxford, Oxford, UK

**Keywords:** Bipolar disorder, Mania, Mental imagery, Cognitive therapy, Goals

## Abstract

**Background:**

Compared to unipolar depression (UD), depressed mood in bipolar disorder (BD) has been associated with amplified negative mental imagery of the future (‘flashforwards’). However, imagery characteristics during positive mood remain poorly explored. We hypothesise first, that unlike UD patients, the most significant positive images of BD patients will be ‘flashforwards’ (rather than past memories). Second, that BD patients will experience more frequent (and more ‘powerful’) positive imagery as compared to verbal thoughts and third, that behavioural activation scores will be predicted by imagery variables in the BD group.

**Methods:**

BD (*n*=26) and UD (*n*=26) patients completed clinical and trait imagery measures followed by an Imagery Interview and a measure of behavioural activation.

**Results:**

Compared to UD, BD patients reported more ‘flashforwards’ compared to past memories and rated their ‘flashforwards’ as more vivid, exciting and pleasurable. Only the BD group found positive imagery more ‘powerful’, (preoccupying, ‘real’ and compelling) as compared to verbal thoughts. Imagery-associated pleasure predicted levels of drive and reward responsiveness in the BD group.

**Limitations:**

A limitation in the study was the retrospective design. Moreover pathological and non-pathological periods of “positive” mood were not distinguished in the BD sample.

**Conclusions:**

This study reveals BD patients experience positive ‘flashforward’ imagery in positive mood, with more intense qualities than UD patients. This could contribute to the amplification of emotional states and goal directed behaviour leading into mania, and differentiate BD from UD.

## 1 Introduction

Bipolar disorder (BD) affects at least 1% of the population (Merikangas et al., 2011; Ten Have et al., 2002), with even higher community estimates (Kodesh et al., 2012; Lewinsohn et al., 2000). It is a leading cause of death due to suicide and associated chronic medical conditions (Merikangas et al., 2007). Despite the burden of the disease and the fact that mood disorders are on a continuum between unipolar and bipolar cases, progress in developing effective therapies for BD lags behind that for unipolar major depression (UD); this is particularly true for psychological interventions (Mansell and Pedley, 2008; Goodwin and Vieta, 2005). Thus, cognitive behavioural therapy (CBT) for BD has not achieved the success seen for UD (Lam et al., 2003, 2005; Scott et al., 2006; Hollon et al., 2005). We have suggested previously that traditional methods of delivering CBT, strongly based on verbal accounts of distorted verbal cognition, may be inappropriate to the distinct cognitive profile of BD (Holmes et al., 2008). We have sought an explanation for this in the growing evidence that clinical assessment and interventions should focus upon the unique role of mental imagery in BD (Holmes et al., 2011; Meyer et al., 2011; Bonsall et al., 2012; Deeprose et al., 2011; Di Simplicio et al., 2012).

Thus, thorough clinical assessment and treatment innovation require an understanding of idiosyncratic spontaneously occurring emotionally charged mental imagery, akin to the flashbacks of PTSD. Whereas the content of PTSD flashbacks is past traumas; we have called certain intrusive imagery in BD as ‘flashforwards’ because it tends to travel forward in time to prospective events. Negative mood states in BD have been associated with intrusive ‘flashforward’ mental images. Hales et al. (2011) interviewed depressed BD or UD individuals who, in line with earlier studies (Holmes et al., 2007b; Crane et al., 2012), described suicide-related images. Both groups reported greater preoccupation with such images than verbal thoughts, but BD individuals were more preoccupied with their imagery and found it more compelling than those with UD. Given that BD has the highest rate of suicide of all psychiatric disorders (Hawton et al., 2005), and imagery promotes action (Libby et al., 2007), we suggested that such compelling suicidal imagery might be highly relevant to clinical risk assessment (Hales et al., 2011; Holmes et al., 2007b).

The present study had three main aims around further understanding imagery in positive mood states in BD: first, to investigate the frequency of ‘flashforwards’ (as compared to past memories) in positive mood states in BD, and to assess the characteristics (e.g. vividness) of such imagery; second, to explore the relationship between behavioural activation levels and positive mental imagery in BD; and finally to contrast these findings with a UD group.

## 2 Methods

We recruited patients with a lifetime BD diagnosis or a lifetime UD diagnosis, from secondary care (outpatients) and the community (advertisements in local community centres and on local and national websites).

### 2.1 Participants

Inclusion criteria for both groups were age 18–65 years; ability to read and write English; and willingness to give written informed consent to participate in the study. Current mood was assessed using the Quick inventory for Depressive Symptomatology Self-Report (QIDS-SR; Rush et al., 2003) and the Young Mania Rating Scale (YMRS; Young et al., 1978).

*Bipolar disorder group*: Lifetime diagnosis of BD type I or II was based on assessment on the Structured Clinical Interview for Diagnostic and statistical manual of mental disorders, 4th Edition (SCID, DSM-IV; First et al., 2002). Exclusion criteria were current episode of mania or hypomania or current severe suicidal ideation, based on the questionnaires above. This resulted in 16 participants recruited with a lifetime history of BD I and 10 with BD II (total=26 BD).

*Unipolar depression group*: Lifetime diagnosis of major depressive episode or dysthymic disorder and no lifetime history of mania or hypomania were based on SCID for DSM-IV (First et al., 2002). Exclusion criteria were current severe suicidal ideation, based on the questionnaire above. This resulted in 23 participants with major depressive disorder and 3 with dysthymic disorder (total=26 UD).

### 2.2 Procedures

Ethical approval for the study was granted by the National Health Service Research Ethics Committee South Central – Oxford A. Written informed consent was obtained from all participants. Those eligible were invited to take part in the interview phase of the study. They completed the Imagery Interview (Holmes et al., 2007a), Beck Anxiety Inventory (Beck and Steer, 1993), Cognition Checklist for Mania – Revised (Beck et al., 2006), Altman Self-Rating Mania Scale (Altman et al., 1997), Impact of Future Events Scale (Deeprose and Holmes, 2010), Spontaneous Use of Imagery Scale (Reisberg et al., 2003) and Behavioural Inhibition/Behavioural Activation Scales (Carver and White, 1994). Participants received £20 for taking part.

### 2.3 Clinical measures and ratings of general imagery use

For detailed description of the measures see Supplementary material.

Quick Inventory of Depressive Symptomatology Self-Report (QIDS-SR; Rush et al., 2003) is a 16-item self-report multiple choice questionnaire measuring depressive depression over the previous seven days.

Altman Self Rating Mania scale (ASRM; Altman et al., 1997) is a 5-item self-report multiple choice questionnaire measuring manic symptoms severity over the previous seven days.

Spontaneous Use of Imagery Scale (SUIS; Reisberg et al., 2003) provides a trait measure of use of (nonemotional) mental imagery in everyday life, for example: “When I think about visiting a relative, I almost always have a clear mental picture of him or her”.

Impact of Future Events Scale (IFES; Deeprose and Holmes, 2010) provides an index of the impact of intrusive imagery of future events over the past seven days.

### 2.4 Endpoint measures

#### 2.4.1 Imagery interview

The Imagery Interview (based on Holmes et al. (2007a)) comprised a modified version of the ‘The Suicidal Cognitions and Flashforwards Interview’ used by Hales et al. (2011) (see also Crane et al., 2012; Day et al., 2004). First, the terms ‘verbal thought’ and ‘mental image’ were explained and the participant’s comprehension checked. Participants were asked to remember and describe a particular point in time when their mood was last ‘positive’, i.e. defined as ‘excited, energised or elevated’. In order to facilitate remembering, a 13-item checklist was used to ascertain the range of content of images and verbal thoughts for the last time of positive mood (Supplementary material, Table S1). The interview then included the following sections.

#### 2.4.2 Most significant positive image

Participants were asked to choose from the checklist the image that they deemed to be the ‘most significant’ and describe it in detail (‘image content’, see Table 3 for BD group and Table S2 for UD group). Participants were asked to rate the characteristics of their selected most significant image including how vivid, exciting and pleasurable it was (from 1=not at all vivid/exciting/pleasurable to 9=extremely vivid/exciting/pleasurable).

#### 2.4.3 Positive imagery versus positive verbal thoughts

Participants were asked to rate the frequency (from 1=not at all to 9=all the time), and ‘powerfulness’ of their imagery. This included preoccupation (from 1=not at all to 9=all the time), how real the images felt (from 1=not at all real to 9=as if it was reality) and how compelling they were (from 1=not at all compelling to 9=completely compelling), for all imagery occurring during the last period of positive mood previously identified. The same ratings were asked separately for all verbal thoughts occurring during the same last period of positive mood.

#### 2.4.4 Measure of behavioural activation

Behavioural Inhibition/Behavioural Activation Scales (BIS/BAS; Carver and White, 1994) are a self-report measure comprising a BIS scale (7 items, such as “Criticism or scolding hurts me quite a bit”) and three BAS scales: Drive (4 items, such as “I go out of my way to get what I want”), Reward Responsiveness (5 items, such as “When good things happen to me, it affects me strongly”) and Fun Seeking (4 items, such as “I am always willing to try something new if I think it will be fun”). Each item is answered using a four point Likert scale, ranging from 1 (strongly disagree) to 4 (strongly agree). These scales have good convergent and discriminant validity (Carver and White, 1994). In an adult sample Cronbach’s *α* values have been reported as 0.77 for the BIS, 0.89 for Reward Responsiveness, 0.85 for Drive and 0.80 for Fun Seeking (Cooper et al., 2007).

### 2.5 Analysis of data

Analyses were carried out using SPSS 17.0 for Windows (SPSS Inc., Chicago, IL, USA). Groups were compared using independent *t*-tests for continuous data and Chi-square tests for categorical data. Within group comparisons were conducted using paired-samples *t*-tests. Effect sizes (Cohen’s *d* for continuous data and Odds Ratio for categorical data) were calculated for all comparisons with the exception of demographic data. Linear regression analyses were run with BIS/BAS scales as dependent factors and clinical group (BD versus UD) and imagery characteristics as independent predictors, to model the relationship between the characteristics of positive mental imagery and behavioural activation traits. A separate regression analysis was conducted for each imagery characteristic investigated. If a significant interaction between clinical group and imagery characteristics was found to predict scores on a behavioural activation scale, this was followed by correlations analysing the relationship between imagery characteristics and behavioural activation within each clinical group separately.

## 3 Results

### 3.1 Demographics, clinical measures and ratings of general imagery use

The sociodemographic, and clinical and general imagery characteristics of the sample are summarised in Tables 1 and 2 respectively. There were no significant between-group sociodemographic differences. The groups were not significantly different on levels of current self-reported depression (mildly to moderately depressed on the QIDS, Rush et al., 2003) and anxiety. The self-rated measures related to mania confirmed that the BD participants were not in a manic state and indeed both groups scored similarly at the time of interviewing. The BD group had a greater number of past illness episodes than the UD group. The rates of co-morbid diagnoses (mostly anxiety disorders for both groups) and the number of unmedicated patients were not significantly different between groups. There was also no difference between the two groups on measures of general imagery use, i.e. the SUIS for trait imagery and the IFES for intrusive prospective imagery (Deeprose and Holmes, 2010; Reisberg et al., 2003).

### 3.2 Endpoint measures

#### 3.2.1 Imagery interview

##### 3.2.1.1 Most significant positive image

Positive ‘flashforwards’ were present across both groups. A summary of the content of each participant’s selected most significant positive image along with the associated affect, meaning and intended behaviour is presented in Table 3 for the BD group and Table S1 for the UD group.

Most significant positive images were categorised as either images of future scenarios (‘flashforwards’) or images of past scenarios (past memories). Two participants in each group described an image without any specific time perspective and were excluded from the analysis. As predicted, there was a statistically significant difference between the two groups in the type of image (‘flashforward’ versus past memory) reported (*χ*^2^=7.38, *P*=0.007, OR=5.9) (Table 4). 83.3% of participants in the BD group described the most significant positive image as a ‘flashforward’, often involving themselves interacting with others, being positively valued or achieving successful goals. These ‘flashforwards’ tended to be appraised as a sign that positive outcomes would actually happen in life and quite strikingly, were often followed by participants’ determination to enact the imagined scenarios (Table 3). In contrast, only 45.8% of participants in the UD group described their most positive significant image as a ‘flashforward’.

In terms of image characteristics, in line with hypotheses, compared to UD, BD participants rated their selected most significant positive image as much more vivid (*t*=2.29, *df*=50, *P*=0.026, *d*=0.65), exciting (*t*=3.40, *df*=49, *P*=0.002, *d*=0.97) and pleasurable (*t*=2.13, *df*=49, *P*=0.040, *d*=0.59) (Table 4).

##### 3.2.1.2 Positive imagery versus positive verbal thoughts

Within BD group, participants with a lifetime history of BD reported significantly more frequent images compared to verbal thoughts (*t*=2.26, *df*=25, *P*=0.033, *d*=0.43). In terms of the ‘powerfulness’ of the imagery and verbal thoughts, higher levels of preoccupation were associated with images compared to thoughts (*t*=2.24, *df*=25, *P*=0.034, *d*=0.070). Images were also rated as significantly more ‘real’ (*t*=2.66, *df*=22, *P*=0.014, *d*=0.73) and more compelling (*t*=3.08, *df*=23, *P*=0.005, *d*=0.77) than thoughts. Within UP group, in contrast, no statistically significant differences were found between images and verbal thoughts within the group of individuals with a life time history of UD (all *P’*s>0.1).

#### 3.2.2 Measures of behavioural activation

Ratings on the BIS/BAS scale measuring behavioural activation showed a significant difference on the ‘Drive’ BAS scale, with the BD group scoring higher than the UD group (BAS Drive: BD, *M*=11.35, SD=2.66; UD, *M*=9.27, SD=2.52; *t*=2.88, df=50, *P*=0.006, *d*=0.80). No differences were present on the other BIS/BAS scales (all *P’*s>0.1).

**Fig. 1:**
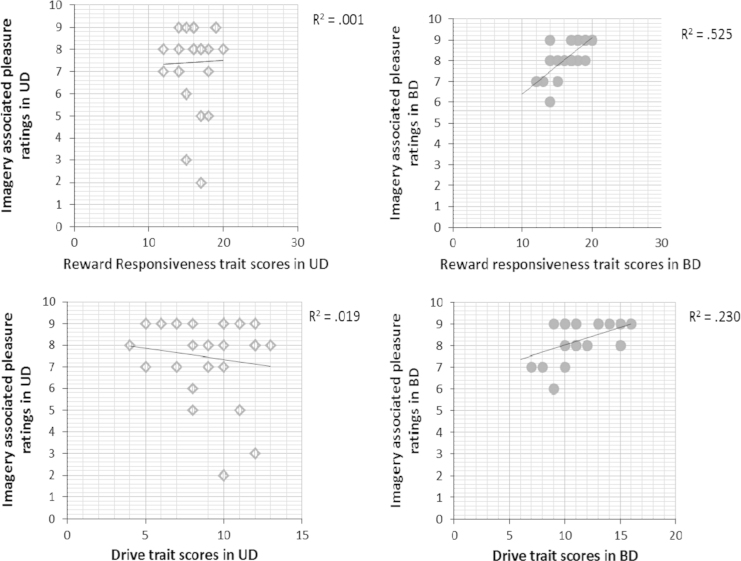
Relationship between behavioural activation trait ratings and characteristics of positive mental imagery in the BD versus UD patients.

To investigate the relationship between BAS scales (‘Drive’, ‘Reward Responsiveness’ and ‘Fun Seeking’), clinical group and image characteristics (e.g. vividness, excitement, pleasure) and power (e.g. realness, compellingness and preoccupation), multiple regressions were performed for each BAS scale with each imagery variable above as one of the predictors, in addition to clinical group (and the interaction between clinical group and imagery characteristic). Only significant regressions are reported. First, in the multiple regression with BAS Drive levels as the dependent variable, and clinical group, imagery-associated pleasure ratings and the interaction between group and pleasure ratings as predictors, the model was significant (*R*^2^=0.278; *df*=3,47, *F*=6.02, *P*=0.001). Both clinical group (BD versus UD) and the interaction between clinical group and pleasure ratings were significant predictors (clinical group: *β*=0.322, *P*=0.020; group×imagery interaction: *β*=0.420, *P*=0.015). Pleasure related to the most significant positive image was significantly positively correlated to BAS Drive in the BD group, (*r*=0.479, *P*=0.015), but not UD group (*r*=−0.136, *P*=0.507) (Fig. 1). Second, the same model was significant when predicting BAS Reward Responsiveness as the dependent variable (*R*^2^=0.310; *df*=3,47; *F*=7.04, *P*=0.001). Image-associated pleasure and the interaction between clinical group and pleasure ratings were significant predictors of Reward Responsiveness (clinical group: *β*=0.322, *P*=0.020; imagery-associated pleasure ratings: *β*=0.682, *P*<0.001, group×imagery interaction: *β*=0.637, *P*<0.001). Pleasure related to the most significant positive image was significantly positively correlated to BAS Reward Responsiveness in the BD (*r*=0.724, *P*<0.001) but not UD group (*r*=0.023, *P*=0.911) (Fig. 1).

## 4 Discussion

This study is the first to explore in detail the frequency and characteristics of positive mental imagery during periods of positive mood across patients with a lifetime history of either BD or UD. As predicted, compared to participants with a history of UD, BD participants reported that their most significant image at times of positive mood was in the form of a ‘flashforward’ (i.e. with a prospective content) rather than a past memory. Moreover, their most significant image at times of positive mood had significantly more intense characteristics, i.e. was more vivid, exciting and pleasurable, compared to the UD group.

When comparing qualities of positive mental imagery to those of positive verbal thoughts during the same time period, BD patients reported a significantly higher frequency of mental images over verbal thoughts (whereas the UD group did not). Further the BD group rated their imagery as more powerful, i.e. more preoccupying, ‘real’ and compelling than their equivalent verbal thoughts. In contrast, the UD group showed no such significant differences between images and verbal thoughts.

BD participants also scored significantly higher on the BAS Drive scale and levels of drive were predicted by levels of pleasure associate to the positive image in BD patients only. BAS Reward Responsiveness was also predicted by pleasure associated with the most significant image in BD patients only.

Overall, our data support the hypothesis that mental imagery in the form of positive ‘flashforwards’ could play an important role in mania. Consistently, among BD participants, future imagery was mostly related to a goal, which promised an attainable, positive experience (Table 3). This is similar to the findings of a previous comparable study (Gregory et al., 2010) in 29 individuals with BD, of whom 66% recalled experiencing intrusive imagery during hypomania (vivid, enjoyable images of future events). A link between hypomania and future imagery has been demonstrated in healthy volunteers at risk for mania BD; “daydreaming” about the future was positively correlated with increased risk for mania (Meyer et al., 2011). We also confirm a disposition of BD patients for prospective mental imagery and further suggest that this might represent a distinctive trait in comparison with UD patients. The positive imagery made our participants want to take literal steps to act on the scenarios they imagined, whether simple as buying a bottle of wine or booking a trip to the Kilimanjaro. This is also in line with Johnson’s model of overly ambitious goal setting as a driver for mania in BD (Johnson, 2005; Johnson and Carver, 2006).

### 4.1 Implications

First, our results could have implications for the understanding of processes involved in bipolar mood instability, i.e. the mechanism by which everyday positive mood could underpin the spiral into pathologically elated states. The differences in mental imagery were not exclusive to periods of pathological positive mood (hypomania or mania). This is consistent with previous data on increased intensity and variability of affective experiences in BD patients even during euthymia (Henry et al., 2008; M’ Bailara et al., 2009; Gruber, 2011). If BD patients habitually present with intense images associated with emotional responses of great pleasure, excitement and preoccupation during euthymic positive mood, this could amplify the positive mood to extreme levels, in line with our previous suggestion (Holmes et al., 2008). For example, one of the patients interviewed described having vivid imagination the idea of a children’s picture book, and of publishing it with a huge success. He felt “complete confidence” that everything would turn out for the better, clearly contributing to the maintenance and possible increase in positive mood. Similarly, another participant reported particularly vivid images of Barcelona (the blue sky and local food smells) that conveyed a feeling of connectedness and being popular among friends, again reinforcing a state of positive affect.

We suspect that these images are so intensely pleasurable that they could account for increased sensitivity to prospective reward. This is unquestionably a central phenomenon of BD together with the bipolar-related biases in the processing of positive emotions (such as self-reported greater positive emotions with greater physiological responses; for a review see Gruber (2011)). It is interesting that fMRI findings have not yet conclusively implicated simple reward pathways (O’sullivan et al., 2011; Caseras et al., 2013; Yip et al., 2012). Future studies could clarify whether frequency and intensity of the ‘flashforwards’ (i.e. vividness, excitement, preoccupation, ‘realness’ and compellingness) grow proportionally to mood elevation in BD and whether imagery is indeed a critical amplifier of subjective experience outside simple anticipation/reward pathways.

Second, our data suggest that alongside mood, ‘flashforwards’ could also impact on behaviour. In the BD group higher imagery-related pleasure appears to predict a higher disposition to spring into action and to respond to rewarding cues measured on the BAS temperamental scales (Alloy et al., 2008). This is likely to enhance goal-directed behaviour (Johnson and Carver, 2006). For example, the images of a weekend in Barcelona reported above pushed the participant to initiate plans for taking a real trip, while the other participant did indeed start writing a children’s book. Future experimental models could confirm the direction and investigate the mechanisms of the association between drive and reward responsiveness, and imagery characteristics. For example, do BD patients experience excessive behavioural activation following the recurrence of pleasant and vivid ‘flashforwards’, as imagining actions increases the likelihood of performing them (Carroll, 1978)?

Overall, we suggest that the impact of amplified ‘flashforward’ imagery on mood and behaviour, possibly combined with high goal-striving traits (Lee et al., 2010) and enhanced processing of positive emotions (Gruber, 2011), holds the potential to accelerate the progress from functional levels of excitement and hyperactivity to full blown manic symptoms.

The findings could also have therapeutic implications. In the earliest CBT models of psychopathology mental imagery was afforded a maintaining role, particularly for anxiety (Beck et al., 1974). However, subsequent decades placed a greater emphasis on verbal thinking, as in challenging “negative automatic thoughts” and keeping “thought records” in CBT for depression (Beck et al., 1979). This is despite experimental evidence demonstrating that imagery elicits more powerful emotional responses than corresponding verbal cognitions (Holmes and Mathews, 2010, 2005). Accordingly, there has been a resurgence of interest in mental imagery (Holmes and Mathews, 2010; Brewin et al., 2010; Holmes et al., 2006, 2008c). Mental imagery has been established as a key driver in posttraumatic stress disorder (PTSD) and social anxiety, with highly successful CBT treatments placing imagery at the centre of therapy (Clark et al., 2006; Ehlers et al., 2005).

Our comparison between imagery and verbal thinking in the BD group suggests, first, that assessment and interventions should target imagery as this bears a more powerful emotional impact on BD patients compared to verbal thoughts. Second, UD patients report less future-oriented positive imagery even during times of positive mood, while showing a focus on the past compared to BD patients. This sits with previous evidence that depressed mood is associated with difficulties engaging in positive prospective imagery (Crane et al., 2012; Morina et al., 2011). Overall, our findings suggest that mental imagery is ‘heightened’ during positive mood in BD (but not UD) individuals. Hence imagery characteristics during positive mood could differentiate UD and BD patients and orientate distinct therapeutic interventions. While UD patients may need training to engage in positive future imagery, BD patients could learn to transform their ‘flashforwards’ towards more balanced characteristics. Further studies could clarify whether imagery characteristics during positive mood also distinguish each clinical group from the healthy population. An additional factor that could be associated with imagery characteristics is the presence and frequency of psychotic episodes. Future studies should record and match clinical groups based on the number of psychotic episodes, in order to investigate if psychotic experiences have an influence on the phenomenology of mental imagery across or within a specific mood disorder.

### 4.2 Limitations

The reliability of the data is limited by its retrospective design. Many participants had to go back significantly into their recent history to find a period when they felt excited or elevated in mood. However, this time gap was equivalent for both clinical groups (median time lapse in BD=70 days, in UD=60 days) and shorter than in previous studies (Gregory et al., 2010). Moreover, self-report of mood elevation is potentially problematic in BD patients, because it is sometimes under-estimated; so it is difficult to discern whether the positive mood episodes reported were of subsyndromal level or not. Therefore, images from pathological and non-pathological periods of positive mood were not distinguished, assuming that the intense imagery characteristics described by our BD sample are related to both.

However, the difference between BD and UD groups is unlikely to be accounted for by response biases due to difference in mood and anxiety at the time of the interview, or by differences in general clinical characteristics such as age at onset, medication or comorbidity; as all of these factors were equivalent in our selected samples. To improve accuracy in defining the affective state of patients at the time of the interview, future studies should also include semi-structured interview measures of depression.

In the absence of a healthy controls comparison group, it cannot be excluded that differences in positive flashforwards characteristics are driven by a negative bias in the UD population. Further studies should then clarify whether BD is characterised by more intense positive mental imagery also relative to the general population. This has implications in terms of the need to design interventions aiming to re-balance positive mental imagery in BD. The findings should also be replicated in a larger sample.

Further, our study was not powered to compare flashforward characteristics between groups of patients receiving different pharmacological treatments. While exploratory investigation indicates no differences in this sample, it cannot be excluded that different pharmacological agents may have an impact on mental imagery phenomenology, similar to their effects on other cognitive processes and this could be addressed in future research.

## 5 Conclusion

In summary, mental imagery has been implicated in the amplification of mood states in mania (Holmes et al., 2008). Extreme imagery features could represent a distinctive hallmark of BD (Holmes et al., 2011; Deeprose et al., 2011, Hales et al., 2011; Malik et al., 2014), and our findings show for the first time that the more intense mental imagery characteristics during positive affect are indeed associated with traits of increased drive to action and greater response to reward in BD compared to UD. If replicated, these data suggest that mental imagery should be given more attention in BD. As current treatments for mania are limited, this emerging area of research could inform new approaches, such as the technique of imagery rescripting used in CBT for anxiety disorders (Holmes et al., 2007a).

Future studies could also investigate the predictive value of positive flashforwards and whether these could distinguish BD from UD patients while being depressed, by using a prospective design including bipolar patients in manic, depressive and remitted phases. Finally a better characterisation of positive mental imagery would include convergent measurements using physiological markers (such as heart rate or blood pressure) associated with the report of intense imagery during the Imagery Interview. This has been shown to be relevant to repetitive intrusive negative imagery in Post-Traumatic Stress Disorder (Orr et al., 1993; Ehlers et al., 2010) and could now be applied to clear repetitive positive and negative intrusive imagery (e.g. self-harm imagery) in BD. Overall, this approach assessing mental imagery could improve clinical assessment of BD with the potential to become a target of pharmacological and psychological interventions alike.

## Role of funding source

EH and MDS are supported by the Medical Research Council (United Kingdom) intramural programme (MC-A060-5PR50). EH is supported by a Wellcome Trust Clinical Fellowship (WT088217), and the National Institute for Health Research (NIHR) Oxford Biomedical Research Centre based at Oxford University Hospitals NHS Trust, Oxford University. GG has received grants from the Medical Research Council and Wellcome Trust.

This research was funded by Oxfordshire and Buckinghamshire Mental Health Trust support to AI, a Wellcome Trust Clinical Fellowship (WT088217) awarded to EH, an Equilibrium Foundation award to MDS and a National Institute for Health Research (NIHR) award under Research Programme Grant for Applied Research (RP-PG-0108-10087) to GG.

The views expressed are those of the author(s) and not necessarily those of the NHS, the NIHR or the Department of Health. Funding to pay the Open Access publication charges for this article was also provided by the UK Medical Research Council.

## Conflict of interest

GG has received honoraria from AstraZeneca, BMS, Eisai, Lundbeck, Sanofi-Aventis and Servier. He has been advisor for AstraZeneca, BMS, Boehringer Ingelheim, Cephalon, Janssen-Cilag, Lilly, Lundbeck, P1Vital, Servier, Shering Plough, Wyeth.

## Figures and Tables

**Table 1:** Sociodemographic characteristics of the sample. Mean (SD – range).

***Demographic characteristics***	***Bipolar*** (*n=26*)	***Unipolar*** (*n=26*)
Age (years)	40.58 (9.91–44)	36.12 (13.06–37)
Female:Male	18:8	18:8

*Ethnicity*		
White British:Other	20:6	18:8
Years in education	14.58 (2.60–9)	14.54 (2.23–9)

*Occupation*		
Employed:not employed	16:10	21:5

*Marital status*		
Single:non-single	16:10	19:7

**Table 2:** Clinical measures and ratings of general imagery use of the sample. Mean (SD).

***Clinical characteristics***	***Bipolar*** (*n*=26)	***Unipolar*** (*n*=26)	***Test***	***p-*****Value**
Depression				
QIDS	9.58 (6.08)	10.88 (5.76)	*t*=0.796	0.430
Mania				
YMRS	1.12 (1.3)	0.27 (0.66)	*t*=2.94	0.006
AMRS	3.27 (3.63)	2.58 (2.67)	U=320.50	0.744
CCLMR total	18.42 (12.35)	18.54 (13.22)	U=329.00	0.869
Anxiety				
BAI	12.86 (10.71)	13.17 (8.47)	*t*=0.113	0.910
Illness variables				
Age at onset	21.72 (9.75)	20.00 (12.11)	U=264.00	0.250
No. of episodes	24.95 (28.73)	4.38 (4.02)	U=53.50	0.001⁎⁎⁎
No. of hospitalisations	2.16 (3.13)	0.46 (0.81)	U=233.50	0.052⁎
Medicated:unmedicated	22:4	19:7	*χ*^2^=1.038	0.308
Lithium	11	0	*χ*^2^=13.95	0.000⁎⁎⁎
Anticonvulsants	11	0	*χ*^2^=13.95	0.000⁎⁎⁎
Antipsychotics	11	2	*χ*^2^=8.30	0.004⁎⁎
Antidepressants	12	19	*χ*^2^=3.91	0.048⁎
SCID				
History of psychosis (yes:no)	12:14	1:25		
Comorbidity:no comorbidity	10:16	14:12	*χ*^2^=1.23	0.266
Body dysmorphic disorder	1	0		
OCD	4	1		
GAD	2	0		
Panic disorder	4	5		
Social phobia	1	2		
Specific phobia	2	1		
PTSD	2	1		
Anorexia nervosa	1	2		
Bulimia nervosa	1	0		
Substance use disorder	1	0		
General imagery measures				
SUIS	43.58 (8.1)	39.88 (9.98)	*t*=1.46	0.15
IFES	35.12 (18.1)	37.62 (14.76)	*t*=0.55	0.59

*Note:* BDI-II=Beck Depression Inventory II, YMRS=Young Mania Rating Scale, QIDS=Quick Inventory of Depressive Symptomatology, BAI=Beck Anxiety Inventory, AMRS=Altman Mania Rating Scale; CCLMR=Cognitive Checklist for Mania – Revised; SUIS=Spontaneous Use of Imagery Scale, IFES=Intrusive Future Events Scale.

⁎ *p*≤0.10 (trend).

⁎⁎ *P*<0.05.

⁎⁎⁎ *P*<0.01.

**Table 3:** Description of the specific image reported by each participant with a lifetime history of bipolar disorder when they last felt excited, energised or elated in mood plus the affect, meaning and intended behaviour reported to be associated with the image.

Participant no. and diagnosis	Content of image	Appraisal of image	Response to image
1. BD I	Someone I’ve nominated being given a knighthood at Buckingham Palace	This is going to happen	To wait for it to happen
2. BD I	A group of people being deeply touched: excited, talkative & bonded by my presence	An affirmation of things I already suspect	I had the image before my birthday, then it was exactly like that, or maybe I just remember it that way
3. BD II	Barcelona: the architecture, blue skies, food smells. A very sociable weekend	Connected to my friends, popular	To ask people if they would be interested in coming to Barcelona
4. BD I	Ski-ing in Switzerland – from packing to arriving and going ski-ing	Learning a new skill is good, it has made me happy	To go ski-ing
5. BD I	Riding my motorbike into a police barricade at the G20 protests, getting injured and being a hero	I can do my bit	Keen to get to the protest
6. BD I	Receiving my assignment, I’m told it was outstanding. Reading it to the group, who congratulate me	Yes, I can do this	To get on with the assignment and achieve this feedback
7. BD II	My 60th birthday, having completed my outdoor art pieces. Strangers appreciating my work	This image will happen	To complete my art pieces and bring the image to life
8. BD II	Ideas for children’s picture book. Publishing it and becoming a huge overnight success	Everything will turn out for the better and it will stay like that	Started work on the book and told people that I was writing it
9. BD II	Private viewing of my sculpture show in New York. People talking, a happy atmosphere	It will happen if I go for it	(None)
10. BD I	Climbing a steep incline up Mount Kilimanjaro. Others walking ahead of me, talking	I want to achieve this, to make up for lost time	To book a trip to Mount Kilimanjaro when I left hospital
11. BD II	Being pushed along on my bicycle, no obstacles: the ride is smooth, fast, easy and natural	What I am doing is right. A built-up tension was being relieved	To take action on things, look for more opportunities and be more adventurous
12. BD I	Graduation: being presented with my qualification. Friends and family being really proud of me	I can achieve this; this is going to be a reality next year	Focus on my studies e.g. my assignments
13. BD I	Christmas Eve with my husband, Christmas Day with my family: how I wanted the day to go	For one day of the year I’ve got all my family and don’t have to share them with anybody	To prepare everything to try and make the image come true
14. BD I	Memory of one Christmas Day. Playing a quiz game, jovial atmosphere, a sense of warmth	I have value, life has value, interacting with others has value	To initiate a game next time
15. BD II	Getting married: walking up the aisle with my father, my fiancée’s face as he turns to look at me	“Everything.” I am loved.	To enjoy the image
16. BD I	My daughter and I working together, her feeling strong, independent, happy, at college	We are going to beat this at whatever cost	Determined that this situation was not going to beat me – to win
17. BD II	My new kitchen, bright and airy, new cupboard doors, modern appliances	Things are going to get better	To start the ball rolling, get started on the renovations
18. BD I	My idea for a sandwich bar at a charity café I had applied to work at	It could be real if I get the job	To say the right things at the job interview, to get the job
19. BD I	Choosing wine to give as a gift to my sisters, imagining the bottles of wine I was going to buy	I want to get this to make them happy	To buy the wine
20. BD I	My hospital room from the doorway looking in: I’m sitting on the bed and trying to be witty	None (“the image was my way of trying to cope”).	Eventually, to take my medication (once I realized my thoughts/imagery were not ‘normal’).
21. BD I	A bird’s flight down to view a beach with a row of buildings, being with my sons	The tranquillity of nature; the unity and harmony of people gathering to do good	I’d like to make it happen; to work towards it, to stay in contact with my sons, to stay with my recovery, to do good things for the world
22. BD I	Viewing my surroundings through a simple kaleidoscope and hearing a broken radio	This is some kind of language	To write it down, but to write it down would spoil it
23. BD I	My brother’s voice, alongside a picture of brown bark on a tree	Knowing that my family are there but also that they’re going	(None)
24. BD II	Memory of skating with my ex-girlfriend. Then in a shopping complex, she saying she likes and admires me	I need to kiss my girlfriend now, I miss my ex, I was fortunate; I’ve had these feelings before	To say to my girlfriend more than “I like you”
25. BD II	Memory of standing on a street with my husband behind me, feeling his hand on my shoulder	This was when I was the happiest	Want to hold on to the image
26. BD II	Memory of a tree-lined lake. Swimming, my children playing on the other side of the lake, feeling a pull towards them	A reminder of the bond between myself and my children	To go and pick my children up and hug them

*Note:* For associated meaning participants were asked, “What did the image mean to you?”, for intended behaviour participants were asked, “What did the image make you want to do?”. BD I=Bipolar I Disorder; BD II=Bipolar II Disorder.

**Table 4:** Qualities of the most significant positive image from a recent period of positive mood (from the Imagery Interview) comparing Bipolar versus Unipolar groups. Values represent frequencies (percentage) for categorical attributes and means (SD) for continuous attributes.

**Variable**	***Bipolar (n=24)***	***Unipolar (n=24)***	***df***	***χ***^***2***^	***p-*****Value**	***OR***
**Most significant positive image**				7.38	0.007⁎⁎⁎	5.9
‘Flashforwards’	20 (83.3%)	11 (45.8%)				
Memories	4 (16.7%)	13 (54.2%)				

	***Bipolar** (n=26)*	***Unipolar (n=26)***	***df***	***t***	***P*****-value**	

Vividness	8.15 (1.04)	7.42 (1.23)	49	2.30	0.026⁎⁎	0.64
Excitement	8.04 (0.97)	6.42 (2.21)	47	3.40	0.002⁎⁎⁎	0.95
Pleasure	8.28 (0.84)	7.42 (1.85)	49	2.13	0.040⁎⁎	0.60

^⁎^*p*≤0.10 (trend).

⁎⁎ *P*<0.05.

⁎⁎⁎ *P*<0.01.
