# Supplementary material for: Mental imagery in bipolar affective disorder versus unipolar depression: Investigating cognitions at times of ‘positive’ mood
Source: J Affect Disord. 2014 Sep;166(100):234–42. doi: 10.1016/j.jad.2014.05.007 (PMC4101244; doi:10.1016/j.jad.2014.05.007)
Supplement: Supplementary file 1 — Supplementary Data [file mmc1.pdf]

# Supplementary Material

## Methods

### *Clinical measures and ratings of general imagery use*

*Quick Inventory of Depressive Symptomatology Self-Report (QIDS-SR; (Rush et al., 2003)).* The QIDS-SR is a 16-item self-report multiple choice questionnaire measuring depressive depression over the previous seven days. On each item (scored 0-3), participants are asked to choose the statement that best describes how they have felt in the past week. Total scores range from 0 to 27. Scores between 6-10 are considered to be indicative of mild depression, between 10-15 of moderate depression and above 15 of severe depression (http://www.ids-qids.org). Rush et al. (Rush et al., 2003) report that the QIDS-SR has high internal consistency (Cronbach's $\alpha$ = 0.86).

*Altman Self Rating Mania scale (ASRM; (Altman et al., 1997)).* The ASRM is a 5-item self-report multiple choice questionnaire that measures the severity of manic symptoms over the previous even days. On each item (scored 0-4), participants are asked to choose the statement that best describes how they have felt in the past week. A cut off score of 6 or higher indicates a high probability of a manic or hypomanic condition. The scale has high internal consistency (Cronbach's $\alpha$ =0.79; 52) and good test-retest reliability (r=0.86; 52).

*Spontaneous Use of Imagery Scale (SUIS*; (Reisberg et al., 2003)). The SUIS provides a trait measure of use of (nonemotional) imagery in everyday life. A series of descriptions are given, for example: ''When I think about visiting a relative, I almost always have a clear mental picture of him or her''. Each description is rated on a 5-point scale, from 1 = *never appropriate* to 5 = *always completely appropriate*.

*Impact of Future Events Scale (IFES*; (Deeprose and Holmes, 2010)). The IFES provides an index of the impact of intrusive prospective imagery measures over the past seven days. Participants complete 24 items to assess intrusive pre-experiencing, avoidance, and hyper-arousal related to

intrusive images of future events. Items include: ''Pictures about the future popped into my mind'' (intrusive pre-experiencing), ''I tried not to think about the future'' (avoidance), and ''I had waves of strong feelings about the future'' (hyperarousal). Each item is anchored on a 5-point scale and a total score is calculated by summation of the responses to the 24 items.

***Endpoint measures***

*Imagery interview*

When recording participants' responses to the Imagery Interview, the duration of time elapsed since the identified period of positive mood was a potential source of bias in participants' accounts. To check whether this was equivalent for both groups, a Mann-Whitney U test was conducted. This confirmed that there was no significant difference between the groups in the number of days since the period of positive mood ($U=294.00$, $p=.72$). The median time lapse was 60 days for the unipolar group and 70 days for the bipolar group.

Participants were asked to remember and describe a particular point in time when their mood was last 'positive', i.e. defined as 'excited, energised or elevated'. In order to facilitate remembering cognitions, first the following 13-item checklist was used to ascertain the range of content of images and verbal thoughts for the last time of positive mood (Table 1S).

**Table 1S**

*The number of participants per group positively endorsing experiences of mental images versus thoughts for each of thirteen categories on the mania-related cognition checklist*

| **Category checklist** | **Bipolar group** (*n*=26) | | **Unipolar group** (*n*=26) | |
|---|---|---|---|---|
| | **Images** | **Verbal** | **Images** | **Verbal** |
| Of yourself planning/ preparing to reach a personal goal | 22 | 22 | 19 | 21 |
| That made you feel excited or motivated | 24 | 21 | 19 | 19 |
| Of you being liked and admired by the people around you | 19 | 13 | 16 | 11 |
| Of an enjoyable time in the past | 20 | 10 | 15 | 8 |
| Of a pleasurable real event that happened in the past | 17 | 12 | 18 | 9 |
| Of you in a powerful position | 15 | 9 | 5 | 3 |
| Of you being full of ideas | 14 | 14 | 14 | 15 |
| Of you being funny and witty, the centre of attention | 15 | 13 | 14 | 9 |
| Of how other people might react if you were to achieve a personal goal | 13 | 15 | 10 | 12 |
| Of what might happen if you were to take a risk and succeed | 12 | 10 | 8 | 12 |
| Of you winning a major award | 3 | 3 | 4 | 3 |
| That were fleeting or unclear | 11 | 9 | 8 | 8 |
| Any other type | 12 | 13 | 12 | 15 |
| Total count *Mean (SD)* | 7.73 (2.78) | 6.35 (3.89) | 6.38 (3.35) | 5.77 (3.08) |

**Results**

**Table 2S**

*Description of the specific image reported by each participant with a lifetime history of unipolar depression when they last felt excited, energised or elated in mood plus the affect, meaning and intended behaviour reported to be associated with the image.*

| Participant no. and diagnosis | Content of image | Appraisal of image | Response to image |
|---|---|---|---|
| 1. MDD | Memory of a party in a marquee. A really colourful disco. In a laughing group of people | Nothing else matters as long as you're laughing and having fun | To go out with my friends and have fun |
| 2. MDD | Memory of taking a client on holiday. The client enjoying himself, me and my wife laughing | It was such a beautiful experience | For the coming day to be a success |
| 3. MDD | Unclear, still image of my cousin the last time I'd seen her at my Grandmother's house | Pleased that I could remember her – as a relative | Nothing |
| 4. MDD | Memory of an event outside my work involving police vehicles, police horses and a news van | Something exciting has happened to me. I've got something important to say | To remember that I want to be in the police force |
| 5. MDD | Memory of being in my old office after making a phone call to bid for a work contract | I've got so much potential. Go for it, it might be easier than I think | To pick up the phone and call people |
| 6. MDD | Memory of my daughter in a Nativity play. Her glittery white dress, sat next to her best friend | My kids are growing up. I'm getting older and I need to get a job | None |
| 7. MDD | Memory of being on a beach with my husband, snorkling.A sense of fun, excitement and fear | There was a time when I was happy – a good experience | Compared what it would be like to do this whilst on holiday now |
| 8. DD | Memory of a walking holiday in Spain: when I had to find my own way back but there was no path | I should have stopped and enjoyed the experience more | To go back and have another go at climbing that mountain, to plan |

| | | | |
|---|---|---|---|
| | | | the trip better |
| 9. MDD | Memories of walking over open countryside with my wife. Meeting my daughter and having lunch | Longing that my life was like how it was in the image all the time | An intention to have more of these times together |
| 10. MDD | Memory of being on a hot beach with my best friend and learning to swim in the still, cold sea | I have succeeded in doing something I wanted to do | To talk about this achievement with my family and best friend |
| 11. MDD | Recurrent memories of specific times spent with my ex-boyfriend | (None) | Longing to be in a relationship with my ex- |
| 12. MDD | Memory of being sat on a bench with three friends watching the world go by | I don't have to worry about these people not understanding me | To hang out more on the bench with these friends |
| 13. DD | Memories of life before my course – empty of ambition or prospects. My future: graduating from University | A symbol of hope | Want to try harder, do better at college and not give up |
| 14. MDD | In my van, the logo of my new business on the side, my Mum and Step-Dad looking proud | In the future my life is going to be better | To speak to my Mum straight away |
| 15. MDD | Reading my literature to an audience, sounds and smells of people eating and drinking | Maybe it will be a success, maybe not | I must finally write this piece out |
| 16. MDD | Role-playing a future telephone call with a work client | I am doing a good job and am using my knowledge and experience well | To deal with the client in a particular way |
| 17. DD | Renovations to my home how I want it to look. It having more light, plus the smell of incense | None | To at least try and work towards this goal |
| 18. MDD | Being professional, business-like in my new job. Visiting various locations, meeting people | I can achieve and be successful in this role | To do the job right away (fleeting desire) |
| 19. MDD | In a beautiful wedding gown on my wedding day, friends and family | This would mean I had achieved my goals, was | To put more effort into |

| | | | |
|---|---|---|---|
| | around, happy, rejoicing | living my dreams | my relationship |
| 20. MDD | Meeting friends at a familiar pub, introducing people. Lots of talking and activity. People smiling | It's going to be fun – it's going to be like this image | Go home sooner! Move back home. Looked at different flights, getting more time off work |
| 21. MDD | At a film institute learning to use a steady-cam, the crew helping me to realise what I want to film | I really want to be in film, to prove others wrong | To get a job, to start saving to be able to attend this film institute |
| 22. MDD | Spending time with a lady friend, physical intimacy, being relaxed and fulfilled, satisfied | This would be really nice, an opportunity to be intimate with someone | To prevent myself fantasising about it |
| 23. MDD | The man I was seeing alone in his office on the first floor of a building | I'm observing him, don't want to be part of it; work is such a big part of his life | None |
| 24. MDD | A lecturer marking my test. An e-mail telling me to meet him. I am told I need to re-take the test | 65% convinced that it would happen. My result must be a mistake | Try to rationalise away my worry |
| 25. MDD | A woodland in early springtime, a feeling of tranquillity and energy, wearing a pale green dress, my partner and other figures | There's now a plan in place, "I'm setting a seal to my future" | To draw pictures, get my ideas on paper and make it reality |
| 26. MDD | In a 1930s 'speakeasy', playing piano for an audience including great piano players of the past | I'm in touch with my inner identity, my creative impulse | To play better – with more intent, purpose and attack |

*Note:* For associated meaning participants were asked, "What did the image mean to you?", for intended behaviour participants were asked, "What did the image make you want to do?". MDD = Major Depressive Disorder; DD = Dysthymic Disorder.
